# Wild Apple-Associated Fungi and Bacteria Compete to Colonize the Larval Gut of an Invasive Wood-Borer *Agrilus mali* in Tianshan Forests

**DOI:** 10.3389/fmicb.2021.743831

**Published:** 2021-10-15

**Authors:** Tohir A. Bozorov, Zokir O. Toshmatov, Gulnaz Kahar, Daoyuan Zhang, Hua Shao, Yusufjon Gafforov

**Affiliations:** ^1^State Key Laboratory of Desert and Oasis Ecology, Xinjiang Institute of Ecology and Geography, Chinese Academy of Sciences, Urumqi, China; ^2^Laboratory of Molecular Biochemistry and Genetics, Institute of Genetics and Plants Experimental Biology, Academy of Sciences of the Republic of Uzbekistan, Tashkent, Uzbekistan; ^3^Laboratory of Mycology, Institute of Botany, Academy of Sciences of the Republic of Uzbekistan, Tashkent, Uzbekistan

**Keywords:** *Agrilus mali*, larval gut microbiota, *Pseudomonas synxantha*, invasive insect, wild apple, antifungal compound

## Abstract

The gut microflora of insects plays important roles throughout their lives. Different foods and geographic locations change gut bacterial communities. The invasive wood-borer *Agrilus mali* causes extensive mortality of wild apple, *Malus sieversii*, which is considered a progenitor of all cultivated apples, in Tianshan forests. Recent analysis showed that the gut microbiota of larvae collected from Tianshan forests showed rich bacterial diversity but the absence of fungal species. In this study, we explored the antagonistic ability of the gut bacteria to address this absence of fungi in the larval gut. The results demonstrated that the gut bacteria were able to selectively inhibit wild apple tree-associated fungi. Among them, *Pseudomonas synxantha* showed strong antagonistic ability, producing antifungal compounds. Using different analytical methods, such as column chromatography, mass spectrometry, HPLC, and NMR, an antifungal compound, phenazine-1-carboxylic acid (PCA), was identified. Activity of the compound was determined by the minimum inhibitory concentration method and electron microscopy. Moreover, our study showed that the gut bacteria could originate from noninfested apple microflora during infestation. Overall, the results showed that in newly invaded locations, *A. mali* larvae changed their gut microbiota and adopted new gut bacteria that prevented fungal colonization in the gut.

## Introduction

Insects have diverse bacterial associations including relationships ranging from parasitism to mutualism (Douglas, 2015). There are a number of studies on the gut microbiota of wood-boring beetles, due to its essential roles in food digestion, compensation for dietary deficiencies, compound detoxification, nutrient production, and more, in the literature (Egert et al., 2003; Douglas, 2015). In addition, insects have a very close relationship with their gut microbiome, and symbiotic interactions can result in host survival under extreme environmental conditions (Gupta and Nair, 2020). There is an unexplored question in invasion ecology, and a study highlighted by Pennisi (2017) raised a new hypothesis: Could the gut microbiome determine the invasion success of phytophagous insects? (Lefort et al., 2017). Recent reports support the crucial role of the gut microflora in insect growth and development and in environmental adaptation (Bahrndorff et al., 2016; Lefort et al., 2017). Gut-associated microorganisms such as bacteria, fungi, protozoa, and viruses can be transiently or permanently transmitted to insects, and this relationship can be either beneficial or harmful (Feldhaar, 2011; Hammer et al., 2017). For example, the gut microflora has a symbiotic relationship with hosts to obtain nutrients, aid digestion, and promote host immunity by protecting against pathogens (Engel and Moran, 2013; Douglas, 2015; Mereghetti et al., 2017). Therefore, studying the microbiome of the insect digestive tract is essential to improve beneficial insect digestive capabilities or pest management programs (Morales-Jiménez et al., 2012).

The interaction between fungi and bacteria in the invasive insect gut has not been thoroughly studied. Fungi and bacteria are frequent in most wood-feeding insect guts and likely play a role in the digestion of plant-cell polymers due to their symbiotic interaction with the host. Microbial composition and abundance in the insect gut vary; wood-feeding insects have a rich bacterial diversity, whereas sap-feeding insects have poor diversity (Engel and Moran, 2013) due to insect gut compartmentalization (Dillon and Dillon, 2004). The bacterial composition of the gut could either be specifically adapted to the gut, maternally transmitted, or acquired from the environment (Ruby et al., 2004). Additionally, the microbial diversity of the gut also depends on the geographic location, which has been shown for honeybees (Yun et al., 2014). The composition of the myco- and microflora of the larval gut are dependent on the developmental stage of the insect and the location, as was shown for the citrus pest *Bactrocera minax* (Yao et al., 2019). However, during larval growth, the gut bacteria may influence fungal diversity (Yun et al., 2014; Sam et al., 2017; Yao et al., 2019). Moreover, gut bacteria can compete with fungi during gut colonization (Sam et al., 2017).

Wild apple *Malus sieversii* (Ledeb.) Roem. is a species native to Tianshan forests and usually found in sub-mountain areas (Lin and Lin, 2000), and it is considered the primary progenitor of all cultivated apples (Richards et al., 2009). Because of its rich genetic diversity, it remains a globally significant genetic resource. Unfortunately, wild apple faces extensive abiotic and biotic stresses. Among them, the invasive insect, a wood-borer *Agrilus mali* (Coleoptera: Buprestidae) has heavily attacked trees since its first detection in the early 1990s and has extensively damaged the wild apple forests of Tianshan (Ji et al., 2004; Bozorov et al., 2019a); since then, 40% of the forest area has been damaged (Wang, 2013). Mainly, mortality of tree is caused by the larval stage of insect that feed cambium, phloem, and outer xylem parts resulting in serpentine galleries that prevent nutrient movement (Ji et al., 2004; Bozorov et al., 2019a). *In situ* or *ex situ* conservation of wild apple *via* propagation nurseries and the development of biotechnological tools to address this problem are necessary.

In our recent study, we showed the microbial diversity of the larval gut of invasive *A. mali* collected from a Tianshan wild apple forest using throughput sequencing and microbiological approaches to detect fungal species. However, the analysis demonstrated the absence of fungal species (Bozorov et al., 2019b). Recently, Zhang et al. (2018) demonstrated that both bacterial and fungal species colonized adult and larval guts of *A. mali* (collected from Ili Kazakh autonomous prefecture, Xinjiang Province, China) when fed different apple species in a laboratory condition. In this study, we explored the origin of *A. mali* larval gut bacteria and their antagonistic interaction with wild apple-associated fungi using microbiological, physiological, and analytical chemistry approaches in the Tianshan Mountain Forest. We hypothesized that the larvae-acquired gut bacteria compete with fungi for colonization in invaded locations. Environmentally acquired bacteria may contribute to shaping the gut microbiome of this important agricultural pest.

## Materials and Methods

### Plant Material Collection and Bacterial Species

The wild apple twigs of randomly chosen trees were collected from the forest in Mohe Village (43°51N, 82°15W), Gongliu County, Ili-Kazakh District, Xinjiang-Uyghur Autonomous Province, China, which is located in the Ili Valley of the Tianshan Mountains. Since insect larvae are specialized in feeding in the phloem/cambium part and rarely in the outer xylem twig, each tree uninfested, larvae-infested, dead twigs, and as well as larval frass were randomly collected. For this purpose, if more than two fourth to fifth instar larvae were found in 50-sm twig (often 1–5 sm in diameter), then it was considered as infested. Moreover, it also considered the healthiness of the infested twig if the upper part of the infested twigs shows more than 50% of dryness. Often, appearance of cracks and coloration change in the bark indicate the presence of larva under it. For twigs that are dried completely but are still on the tree, we considered them as dead twigs. Larval frass was collected from the respective collected infested twig.

The gut bacterial species [*Pseudomonas synxantha* (#283), *Ps. orientalis (#24)*, *Erwinia billingiae (#32)*, *E. persicina (#12)*, and four *Pantoea species strains (#2, 43, 153, 287)*] were used from the bacterial cryopreserved stock from the previous study (Bozorov et al., 2019b). The bacteria were grown in Luria–Bertani broth medium (Sigma).

### Isolation of Apple-Associated Fungi and Bacteria

Five replicates of uninfested, infested, and dead wild apple twigs, as well as larval frass were used to isolate fungi and bacteria. Larval frass was collected from serpentine galleries made by larvae after removal of the twig bark. Next, healthy, infested, and dead twigs with xylem, bark, and frass were ground with a home blender in sterile conditions. Ground tissues were placed on the respective PDA medium (potato starch 4 gm L^–1^, dextrose 20 gm L^–1^, and agar 15 gm L^–1^, pH 5.6) (Potato Dextrose Agar, Solarbio, P8931-250G) and nutrient agar (NA) (0.5% peptone, 0.3% beef extract, 1.5% agar, pH 6.8) (Difco, France) for fungi and bacteria isolation, and incubated at 25°C. Single colonies were isolated and re-cultivated to classify their morphological features.

### DNA Extraction

Fungal isolates were cultivated on PDA medium for 7–14 days upon sufficient production of mycelia. Mini-preparation of fungal DNA method (Liu et al., 2000) with minor modification was used to extract the fungal DNA. Briefly, the cell walls of fungi mycelia were ground with mortar and pestle in the presence of liquid nitrogen. One milliliter of lysis buffer [400 mM Tris-HCl (pH 8.0), 60 mM EDTA (pH 8.0), 150 mM NaCl, 1% sodium dodecyl sulfate] was added into the fine powdered fungal mycelia. Next, the mix was transferred into a 2-ml Eppendorf tube, and thoroughly mixed, and left at room temperature for 10 min. Then, 0.3 ml of potassium acetate (pH 4.8; which is made of 60 ml of 5 M potassium acetate, 11.5 ml of glacial acetic acid, and 28.5 ml of distilled water) was added, vortexed briefly, and centrifuged at 10,000 × *g* for 1 min. The supernatant was transferred into a new 2-ml Eppendorf tube, and an equal volume of isopropyl alcohol was added and mixed by inversion. Tubes were centrifuged at 10,000 *g* for 2 min, and the supernatant was discarded. Pellet was washed with 0.3 ml of 70% ethanol and was spun at 10,000 × *g* for 1 min, then the supernatant was discarded. The DNA pellet was air dried and dissolved in 50 ml of 1 × Tris-EDTA buffer. Bacteria DNA extraction was carried out following our earlier study (Bozorov et al., 2019b).

### PCR Analysis and Sequence Analysis

For apple-associated fungi isolate identification, the internal transcribed spacer (ITS) region was amplified using primer pairs ITS1 (5′-TCCGTAGGTGAACCTGCGG-3′) and ITS4 (5′-TCCTCCGCTTATTGATATGC-3′) (White et al., 1990). Amplifications were performed in a total volume of 50 μl containing 10 μl of PrimeSTAR HS (Premix) (Takara, Japan) with an appropriate concentration of dNTPs (0.2 mM) and Taq polymerase (5 U), 1 μl (0.2 μM) of each primer, and 1 μl of diluted DNA. The PCR conditions included 5 min at 95°C for the initial step followed by 35 cycles at 94°C for 15 s (denaturation), 55°C for 30 s (annealing), and 72°C for 2 min (elongation), with a final extension at 72°C for 10 min. PCR products were visualized on a 1.0% agarose gel. PCR products were sequenced bidirectionally with the Sanger method in Beijing Genomics Institute (Shenzhen, China).

PCR amplification of 20-fold-diluted bacterial DNA was performed on a Veriti thermocycler (Applied Biosystems, United States) using forward 27F 5′-AGAGTTTGATCATGG CTCAG-3′ and reverse 1492R 5′-TACGGCTACCTTGTTA CGACTT-3′ primers (Broderick et al., 2004). PCR reaction and condition were performed following our earlier study (Bozorov et al., 2019b).

Sequences were assembled using SeqMan (DNASTAR Lasergene 7). Sequences of ITS and 16S RNA were compared with respective other orthologous fungal and bacterial sequences deposited in GenBank using the BLASTN algorithm. Representative OTUs and sequences from the Sanger method were aligned with CLUSTALW. Maximum likelihood (ML) phylogenetic tree was constructed based on the neighbor-joining algorithm following the Tajima–Nei model with 1,000 bootstrap replicates in MEGA7.

### Extraction and Purification of Antifungal Compounds

Each gut bacteria was cultured in 2-L Erlenmayer flasks with 5 L of LB liquid medium. After 5 days, the culture was centrifuged at 8,000 rpm for 10 min to obtain a cell-free supernatant. Supernatants were dehydrated under a fume hood. Initially, a small part of the dried content was divided into three parts and dissolved in either petroleum ether, dichlormethane, or methanol to determine the efficient extracting solvent for antifungal compound. Next, these extracts were vortexed and centrifuged at 10,000 rpm for 5 min. Supernatants were concentrated with a rotary evaporator, and contents were dissolved in 1 ml of the appropriate solution and was examined for its antifungal ability using agar diffusion assay against selected fungi.

The rest of the dehydrated supernatant was extracted with dichlormethane. Dichlormethane phase was concentrated using a rotary evaporator (IKA RV8V, Germany). Crude extract was fractionated with silica gel or sephadex columns chromatography. The crude extract was mixed with an equal mass of silica gel (200–300 mesh) (Qingdao Marine Chemical Company, China), mixed, and loaded on top of the chromatography column (80 cm length and 5 cm diameter) containing 280 g of silica gel. The chromatography column was washed with a mobile phase (v/v) with different proportions of petroleum ether: methanol (100:0, 36:1, 18:1, 9:1, 4:1, 2:1, 1:1, and 0:100 v/v) and ethyl actetate: methanol (9:1, 4:1, 2:1, 1:1, and 0:1 v/v). About 7 ml of eluate was collected in glass vials, monitored with thin layer chromatography (TLC), and each fraction was examined for antifungal activity by agar diffusion. Active fractions were combined based on TLC and diffusion agar results and concentrated using the rotary evaporator. Next, combined fractions were loaded on to a Sephadex column (50 cm length and 1.5 cm diameter). The Sephadex column (Sephadex LH-20, Amersham Pharmacia Biotech, Sweden) was washed with a mobile phase of chloroform: methanol proportion (1:1 v/v). Fractions with 7 ml were collected to glass vials and monitored with TLC. Antifungal activity was examined by agar diffusion against fungi. Next, based on TLC and activity results, active fractions were combined and re-extracted again by silica gel column. Furthermore, the column was washed with a mobile phase with different proportions of hexane: ethyl acetate (100:0, 40:1, 30:1, 20:1, 15:1, 12:1, 10:1, and 0:100 v/v). The fractions were examined by TLC and for antifungal activity. Positive fractions were purified with a Sephadex column by washing with different proportions of dichlormethane:methanol mobile phase (100:0, 70:1, and 0:100).

### Thin Layer Chromatography, HPLC, Mass Spectrometry, and NMR Analyses

To investigate the qualitative compositions of the antifungal compound, TLC was applied. TLC analysis was used to monitor the fractions from column chromatography, and spots on silica gel plates were visualized by spraying with a solution (1.5% of aluminum chloride in ethyl alcohol, ammonia vapor, and 5% sulfuric acid in ethyl alcohol pre-heated at 105°C). Next, purity of compound was measured by an UV spectrophotometer (UV-2550 Shimadzu, Japan).

HPLC analysis was performed using a Hitachi Chromaster HPLC system consisting of an 1,110 pump, DT-230 column oven, 1,430 diode array detector, and a YMC C18 column (250 × 4.6 mm, 5 μm). HPLC analysis was performed with the EZChrom Elite software. The mobile phases were water, acetonitrile, and methanol. The mass spectra were measured in a 2690-ZQ 4000 Water-Alliance LC-MS spectrometer (Applied Biosystems/MDS Sciex Concord, ON, Canada). ^1^H NMR, ^13^C NMR, and 2D NMR spectra were recorded on Varian MR-400 and VNMRS-600 NMR spectrometers with TMS as an internal standard.

### Agar Diffusion

To determine the antagonistic abilities of the gut bacteria, a mix of half of the ISP2 medium (yeast extract 4 g L^–1^, malt extract 10 g L^–1^, dextrose 4 g L^–1^, and agar 20 g L^–1^, pH7.2) and half of the PDA were used to pour onto a plastic petri dish (90 cm in diameter). Next, each gut bacteria was co-cultured with different fungi isolates. Co-cultivation experiments in single plate was repeated, and the antagonistic ability of bacteria was determined by evaluation of distance between the bacterial growth edge (from the fungal side) and the fungi growth edge (from the bacterial side). Inhibition of fungal growth was calculated by using the following equation (Alenezi et al., 2016):


I(%)=(1-a/b)×100

where *a* is the distance from the center of the fungal colony to the fungi growth edge (from the bacterial side and bacterial growth edge), and *b* is the radius of control of the fungal colony.

To examine antifungal activity of the chromatography fractions, 3-mm diameter holes were punched out in PDA plates with a hole puncher. Fractions from each column chromatography were loaded into a well under sterile flow cabinet. A 5-mm piece of fungi mycelium grown on PDA was punched out and transferred onto the middle of the PDA plate. The plates were cultured for 7 days at 25°C, and fungi growth was recorded every day post cultivation.

### Determining Enzymatic Activities

To evaluate different enzyme activities of lignocellulolytic pathways that are involved in the degradation of plant cell wall compounds such as cellulose, lignin, glucans, cellobiose, and xylan, for cellulose degradation, steps were performed following the report by Vasanthakumar et al. (2008). For ligninolytic activity and lignin oxidation assay, reports from Machado et al. (2005) and Vasanthakumar et al. (2008) were followed. Activities of xylanase, cellobiase, and glucanase were determined by evaluation of the coloration of the respective substrates (Vargas-Asensio et al., 2014; Rojas-Jiménez and Hernández, 2015). For lipolytic and proteolytic activities, respective tween 20/80 and milk powder were used as substrates. Enzyme activities of the gut bacteria were evaluated by the appearance of clear halos (Mayerhof et al., 1973; Kumar et al., 2012).

### Minimum Inhibitory Concentration

The MIC for selected fungi were determined using a 10-fold serial dilution method. Petri dishes with PDA medium were prepared. Diluted pure compound with respective concentrations (20, 10, 5, 2.5, 1.25, 0.63, 0.31, 0.16, and 0.08 μg) was mixed with the PDA medium. The punched-out fungi mycelium with 0.7-mm diameter gel piece was transferred onto the middle of the agar plate, and mycelial growth was evaluated after 3, 6, 8, 10, and 12 days of post incubation at 25°C. Each treatment was performed in triplicate, and the diameter of the mycelial growth inhibition (MGI) was measured with a caliper, and calculated according to the following equation:


$$\mathrm{MGI}\left(\%\right)=\left[\frac{\text{Dc}-\text{Dt}}{\text{Dc}}\right]\times 100$$

where, Dc (mm) is the mean of the colony diameter in the control, and Dt (mm) is the mean of the colony diameter in each treatment.

### Scanning Electron Microscopy

To examine the effect of the antifungal compound on the fungi hyphae structure, the wild apple-associated *Dothiorella sarmentorum* fungi was selected. The aerial mycelium of the fungi, a glass coverslip was placed on the surface of the PDA medium inoculated with a mycelial plug and cultured for 4, 6, or 12 days. Glass coverslips covered with mycelium that were directly coated with platinum for 120 s by an Iron Sputter Coater E1045 (Hitachi, Japan) and were viewed under a scanning electron microscope (Carl Zeiss Jena, SUPRA 55VP, Germany) as described earlier in our study (Liu et al., 2020).

### Statistical Analysis

All the experimental data consisted of the means of at least three independent replicates, and comparisons of data were performed using one-way ANOVA with Fisher’s PLSD *post-hoc* test. A *p-*value of <0.05 was considered statistically significant. All statistical analyses were performed using StatView software packages (SAS Institute Inc., Cary, NC, United States). All figures were generated in Adobe Illustrator CS3 Version 13.0.0.

## Results

### Identification of Cultivable Wild Apple-Associated Fungi and Bacteria

**FIGURE 1:**
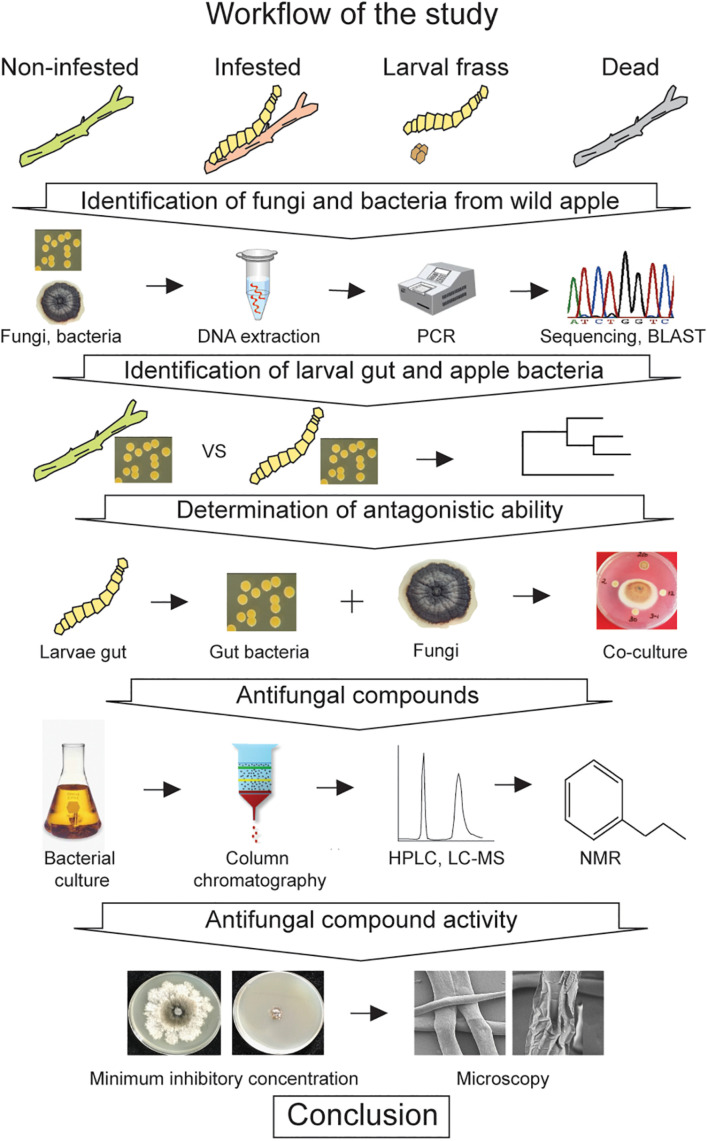
A workflow depicting the strategy used to investigate the gut microbiota against the wild apple-associated fungi.

To elucidate competition between wild apple-associated fungi and bacteria in terms of the larval gut of *A. mali* colonization, several molecular, microbiological, and analytical tools were applied. For this purpose, we developed an exploratory strategy to conduct stepwise identification of the host tree-associated microorganisms and screen the gut bacteria for antagonistic ability as well as determine the origin of the gut bacteria, as depicted in Figure 1.

Based on the workflow, we first isolated the fungi and bacteria from different states of twigs, such as noninfested, larvae-infested, and dead twigs, as well as larval frass, to obtain as many species as possible, since the *A. mali* larvae especially the *Malus* species feeds on twigs, especially in the younger twigs. A total of 204 monoconidial fungi and 320 bacterial isolates were obtained. Each fungus and bacterium were individually isolated. Individual DNA was extracted from each fungal and bacterial colony. For preliminary identification of fungal and bacterial isolates, the respective internal transcribed spacer (ITS) and 16S RNA regions were amplified. The traditional Sanger method was performed to sequence the regions. Next, sequences of host tree-associated bacterial isolates and sequences of the gut bacteria from our earlier study (Bozorov et al., 2019b) were compared to understand the origin of the gut bacteria. Then, the antifungal activity of the gut bacteria against the host tree-associated fungi was determined. Antifungal compounds were isolated from the strongest gut bacteria, which were hypothesized to prevent fungal growth in the gut. Furthermore, the activity of the antifungal compounds was analyzed.

**FIGURE 2:**
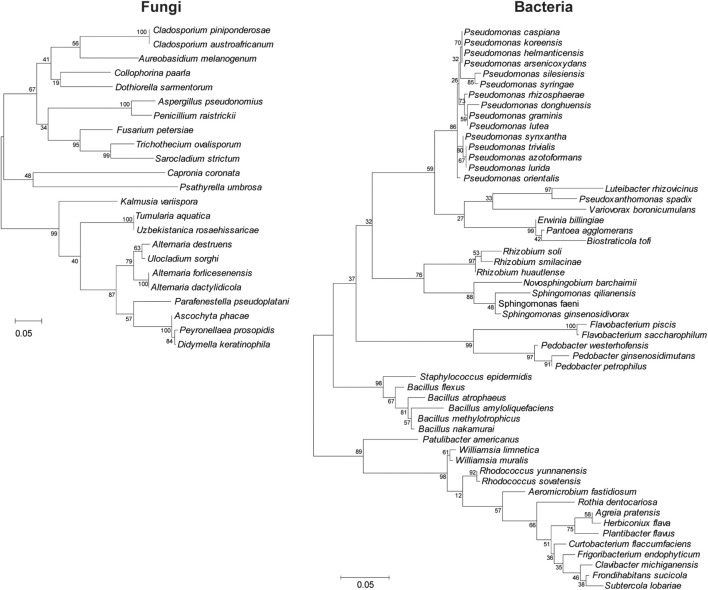
Clustering analysis of the wild apple-associated fungal and bacterial diversity. Maximum likelihood was inferred using the neighbor-joining method. The percentage of replicate trees in which the associated taxa clustered together in the bootstrap test (1,000 replicates) is shown next to the branches. The evolutionary distances were computed using the Tajima–Nei method and are in units of the number of base substitutions per site. Distance scale represents the number of differences between the sequences. Evolutionary analyses were conducted in MEGA 7.

Sequence analysis among the 204 culturable fungal and 320 bacterial isolates demonstrated 23 and 54 operational taxonomic units (OTUs), respectively (Figure 2). Fungal isolates were distributed into two phyla, seven classes, 13 orders, 15 families, and 19 genera, whereas bacterial isolates corresponded to four phyla, eight classes, 14 orders, 17 families, and 27 genera (Figure 2 and Tables 1, 2).

**TABLE 1:** Identified fungal isolates from wild apple stems by internal transcribed spacer (ITS) sequence analysis and their taxonomic status.

Phylum	Class	Order	Family	Genus	Predicted species	GenBank accession	Number of isolates
Ascomycota	Chaetothyriomycetidae	Chaetothyriales	Herpotrichiellaceae	*Capronia*	*Ca. coronata*	NR_154745***	1
	Dothideomycetes	Pleosporales	Pleosporaceae	*Alternaria*	*A. dactylidicola*	NR_151852*	1
					*A. destruens*	NR_137143*	83
					*A. forlicesenensis*	NR_151853*	2
					*A. sorghi*	NR_160246*	1
			Melanommataceae	*Uzbekistanica*	*U. rosae-hissaricae*	NR_157549*	5
		*Not ranked*	*Not ranked*	*Tumularia*	*T. aquatica*	NR_145347**	8
	Eurotiomycetes	Eurotiales	Trichocomaceae	*Aspergillus*	*As. pseudonomius*	NR_137444*	1
				*Penicillium*	*P. raistrickii*	NR_119493*	4
	Leotiomycetes	Phacidiales	*Not ranked*	*Pallidophorina*	*Pa. paarla*	NR_119749*	6
	Sordariomycetes	Hypocreales	Sarocladiaceae	*Sarocladium*	*S. strictum*	NR_111145*	1
			Nectriaceae	*Fusarium*	*F. petersiae*	NR_156397*	4
			*Not ranked*	*Trichothecium*	*T. ovalisporum*	NR_111321*	1
		Dothideales	Dothioraceae	*Aureobasidium*	*Au. melanogenum*	NR_159598*	2
		Pleosporales	Didymellaceae	*Ascochyta*	*As. phacae*	NR_135942*	3
				*Didymella*	*D. keratinophila*	NR_158275*	36
		Capnodiales	Davidiellaceae	*Cladosporium*	*C. austroafricanum*	NR_152288*	1
					*C. pini-ponderosae*	NR_119730*	1
				*Peyronellaea*	*Pe. prosopidis*	NR_137836*	29
			Montagnulaceae	*Kalmusia*	*K. variispora*	NR_145165*	4
		Pleosporineae	Cucurbitariaceae	*Parafenestella*	*Par. pseudoplatani*	NR_165542*	8
		Botryosphaeriales	Botryosphaeriaceae	*Dothiorella*	*Do. sarmentorum*	NR_111166*	1
Basidiomycota	Agaricomycetes	Agaricales	Psathyrellaceae	*Psathyrella*	*Ps. umbrosa*	NR_161031***	1

Asterisks indicate *>97%, **>95%, and ***>85% similarities.

**TABLE 2:** Identified bacterial isolates from wild apple stems by 16S RNA sequence analysis and their taxonomic status.

Phylum	Class	Order	Family	Genus	Predicted species	GenBank accession	Number of isolates
Actinobacteria	Actinobacteria	Actinomycetales	Nocardioidaceae	*Aeromicrobium*	*Ae. fastidiosum*	NR_044983.2*	9
		Corynebacteriales	Nocardiaceae	*Rhodococcus*	*Ro. sovatensis*	NR_156055.1*	1
					*Ro. yunnanensis*	NR_043009.1*	3
				*Williamsia*	*W. limnetica*	NR_117925.1*	3
					*W. muralis*	NR_037083.1**	1
		Micrococcales	Microbacteriaceae	*Agreia*	*A. pratensis*	NR_025460.2*	5
				*Clavibacter*	*Cl. michiganensis*	NR_134712.1*	1
				*Curtobacterium*	*Cu. flaccumfaciens*	NR_025467.1*	25
				*Frigoribacterium*	*Fr. endophyticum*	NR_134732.1****	2
				*Frondihabitans*	*Fh. sucicola*	NR_125644.1**	6
				*Herbiconiux*	*H. flava*	NR_113225.1**	4
				*Plantibacter*	*P. flavus*	NR_025462.1*	1
				*Rothia*	*R. dentocariosa*	NR_074568.1*	1
				*Subtercola*	*S. lobariae*	NR_156868.1***	1
	Thermoleophilia	Solirubrobacterales	Patulibacteraceae	*Patulibacter*	*Pa. americanus*	NR_042369.1*	3
Firmicutes	Bacilli	Bacillales	Bacillaceae	*Bacillus*	*B. amyloliquefaciens*	NR_117946.1**	1
					*B. atrophaeus*	NR_024689.1**	1
					*B. flexus*	NR_113800.1*	1
					*B. methylotrophicus*	NR_116240.1*	15
					*B. nakamurai*	NR_151897.1*	1
			Staphylococcaceae	*Staphylococcus*	*St. epidermidis*	NR_036904.1*	1
Bacteroidetes	Flavobacteriia	Flavobacteriales	Flavobacteriaceae	*Flavobacterium*	*F. piscis*	NR_133746.1*	1
					*F. saccharophilum*	NR_112839.1*	4
	Sphingobacteriia	Sphingobacteriales	Sphingobacteriaceae	*Pedobacter*	*Pe. ginsenosidimutans*	NR_108685.1*	2
					*Pe. petrophilus*	NR_156885.1*	1
					*Pe. westerhofensis*	NR_042602.1*	2
Proteobacteria	Alphaproteobacteria	Rhizobiales	Rhizobiaceae	*Rhizobium*	*Rh. huautlense*	NR_024863.1**	4
					*Rh. smilacinae*	NR_148270.1*	3
					*Rh. soli*	NR_115996.1*	4
		Sphingomonadales	Sphingomonadaceae	*Novosphingobium*	*N. barchaimii*	NR_118314.1*	1
				*Sphingomonas*	*Sp. faeni*	NR_042129.1**	2
					*Sp. ginsenosidivorax*	NR_117830.1**	1
					*Sp. qilianensis*	NR_146363.1*	1
	Betaproteobacteria	Burkholderiales	Comamonadaceae	*Variovorax*	*V. boronicumulans*	NR_114214.1*	1
	Gammaproteobacteria	Enterobacterales	Erwiniaceae	*Erwinia*	*E. billingiae*	NR_104932.1*	30
				*Pantoea*	*P. agglomerans*	NR_041978.1*	9
			Pectobacteriaceae	*Biostraticola*	*Bi. tofi*	NR_042650.1***	1
		Pseudomonadales	Pseudomonadaceae	*Pseudomonas*	*Ps. arsenicoxydans*	NR_117022.1*	19
					*Ps. azotoformans*	NR_113600.1*	3
					*Ps. caspiana*	NR_152639.1*	13
					*Ps. donghuensis*	NR_136501.2**	11
					*Ps. graminis*	NR_026395.1*	36
					*Ps. helmanticensis*	NR_126220.1*	5
					*Ps. koreensis*	NR_025228.1*	3
					*Ps. lurida*	NR_042199.1	23
					*Ps. lutea*	NR_029103.1*	10
					*Ps. orientalis*	NR_024909.1*	5
					*Ps. rhizosphaerae*	NR_029063.1**	2
					*Ps. silesiensis*	NR_156815.1*	5
					*Ps. synxantha*	NR_113583.1*	1
					*Ps. syringae*	NR_074597.1**	8
					*Ps. trivialis*	NR_028987.1*	8
		Xanthomonadales	Rhodanobacteraceae	*Luteibacter*	*L. rhizovicinus*	NR_042197.1*	7
			Xanthomonadaceae	*Pseudoxanthomonas*	*Px. spadix*	NR_042580.1**	8

Asterisks indicate *>99%, **>98%, ***>97, and ****>96% similarities.

**FIGURE 3:**
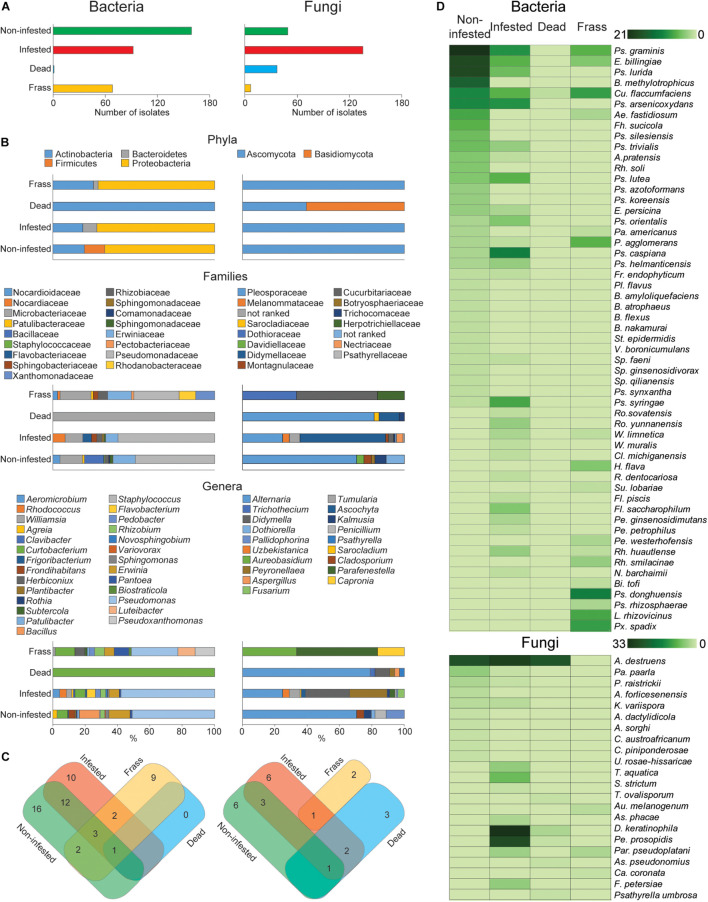
Fungal and bacterial diversity and abundance in different wild apple twigs and larval frass. **(A)** Number of isolates of fungi and bacteria of different twigs and larval frass. **(B)** Percentage distribution of isolates by their phylum, family, and genus. **(C)** Venn diagram summarizing the composition of fungal and bacterial isolates at the species level. **(D)** Heat map analysis displaying a comparison of fungal and bacterial abundance by different twigs and larval frass.

The abundance and composition of the apple twig fungi and bacteria differed depending on the material categories (Figure 3). The abundance of fungal isolates was increased in infested twigs compared with non-infested and dead twigs, whereas bacterial diversity was richer in non-infested twigs than in either infested or dead twigs. Additionally, the number of fungi and bacterial isolates in larval frass also differed from the other material categories (Figure 3A). Fungi rather than bacteria mostly colonized dead twigs, whereas a higher abundance of bacterial isolates was found in the larval frass.

The composition of isolates demonstrated that the fungi and bacteria can colonize twigs commonly or specifically. The distribution of isolates by phylum, family, and genus revealed diverse colonization of different twigs and larval frass (Figure 3B). For example, an additional fungal phylum, Basidiomycota, was found in dead twigs, whereas bacteria were reduced to one phylum. This suggests that increased saprophytic fungi such as *Alternaria* and *Psathyrella* in dead twigs could antagonistically reduce bacterial species.

Larval attack made a disappearance of the bacterial phylum Firmicutes but emerged Bacteroidetes. Similarly, it was observed in larval frass (Figure 3B). The Bacillaceae, Staphylococcaceae, and Comamonadaceae families were specific to non-infested twigs, whereas, the Flavobacteriaceae, Nocardiaceae, and Sphingobacteriaceae families were specific to infested twigs, and the Pectobacteriaceae, Rhodanobacteraceae, and Xanthomonadaceae were specific to the larval frass. At the genus level, *Frondihabitans*, *Plantibacter*, *Bacillus*, *Staphylococcus*, *Sphingomonas*, and *Variovorax* were replaced by *Rhodococcus*, *Clavibacter*, *Williamsia*, *Rothia*, *Flavobacterium*, *Pedobacter*, and *Novosphingobium* in the infested twigs and *Luteibacter*, *Pseudoxanthomonas*, *Biostraticola*, *Subtercola*, and *Herbiconiux* in the larval frass. Dead twigs showed only one species, *Curtobacterium flaccumfaciens*, with the lowest abundance (Figure 3B).

The distribution of fungal species by their taxonomy differed from that of bacteria. The genera *Cladosporium* and *Dothiorella* specifically colonized noninfested twigs while *Uzbekistanica*, *Tumularia*, *Sarocladium*, *Ascochyta*, *Didymella*, *Peyronellaea*, and *Fusarium* were detected from infested twigs. *Trichothecium* and *Psathyrella* as well as *Aureobasidium* and *Capronia* were specific to dead twigs and larval frass, respectively. Distribution by species composition of fungi and bacteria revealed that 16 bacterial species were specific to noninfested twigs, 10 to infested twigs, and nine to larval frass, whereas six fungal species were specific to noninfested twigs, five to infested twigs, three to dead twigs, and two to larval frass. Taken together, during larval infestation, host tree-associated bacterial composition was reduced, whereas fungal species were increased.

### Determining Antagonistic Ability of Larval Microflora

**FIGURE 4:**
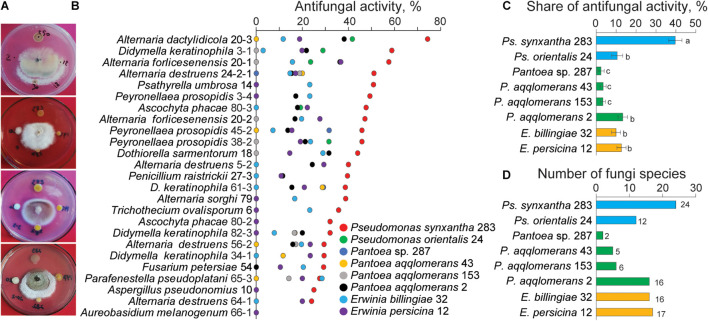
Antifungal activity of the gut bacteria against different fungal species isolated from wild apple twigs. **(A)** Examples of the gut bacterial inhibition of wild apple fungi. **(B)** Antifungal activity of the gut bacteria against selected fungi from the different types of apple twigs. **(C)** Share of antifungal activity of each gut bacteria. Different letters indicate significant differences determined by one-way ANOVA, followed by a Fisher PLSD *post-hoc* test (*p* < 0.05). **(D)** Number of fungal isolates inhibited by each gut bacterium.

In our previous study, we demonstrated the rich diversity of the *Agrilus mali* larval gut microbiota, and approximately 99% of the taxa were cultivable bacteria (Bozorov et al., 2019b). To determine the antagonistic ability of the larval gut bacteria toward apple-associated fungi, *in vitro* screening was performed using cocultivation (Figure 4A). For this experiment, 25 fungi belonging to different species isolated from apple twigs were randomly selected. The results indicated that all the gut bacteria demonstrated antagonistic ability in a selective manner (Figure 4B). The distribution of the share of total antifungal activity by each gut bacteria differed. Among them, *Ps. synxantha* was able to inhibit fungal isolates with a 41.8% share (24 fungal species), *Ps. orientalis* with an 11.0% share (12 fungal species), *E. billingiae* with a 10.4% share (16 fungal species), *E. persicina* with a 13.3% share (17 fungal species), and *Pantoea* sp. with 2.5% share (two fungi species). Three *P. aqqlomerans* strains, 2, 153, and 43, inhibited fungi with a 14.0% (16 fungal species), 3.5% (six fungal species), and 3.5% share (five fungal species), respectively (Figures 4C,D). The results suggest that *Ps*. *synxantha* was the strongest antagonist in the larval gut and was able to inhibit 90.9% of the selected host tree-associated fungal species. The difference in antifungal activity of bacteria could be due to the level of fungi ability to neutralize the effect of bacterial toxins and the level of bacterial compound.

### Determination of Enzymatic Activities of the Gut Bacteria

The gut bacteria perform various enzymatic functions in the degradation of cell wall compounds, lipids, and proteins (Table 3). In a previous report, *A. mali* larvae gut collected from wild apple twigs represented 99.1% of cultivable *Pantoea*, *Erwinia*, and *Pseudomonas* species. Other 36 OTUs were non-culturable bacteria (Bozorov et al., 2019a). We showed that only *Pantoea*, *Erwinia*, and *Pseudomonas* species consisted of the examined ability of the gut bacteria to degrade plant cell wall components, but in this study, we showed the levels of bacterial lysocellulotic and other enzymatic activities. *Pantoea* species were able to highly degrade lignocellulosics such as cellulose, xylanase, glucanase, and cellobiose but not lignin, lipids, or proteins. Both *Erwinia* species showed weak cellulolytic activity, and only *E. persicina* demonstrated xylanase, glucanase, and lipase activities. *Pseudomonas* species were not able to degrade plant cell wall compounds but showed strong protease activity. Moreover, *Ps. orientalis* showed weak lignocellulolytic and strong lipolytic activities. Lignin oxidation was not observed by any gut bacteria.

**TABLE 3:** Enzymatic activities of the gut bacteria from insect larvae.

Species	Cellulose	Xylanase	Glucanase	Cellobiase	RBBR^a^	Protease	Lipase	LAC
*Erwinia billingiae* 32	+	−	−	−	−	−	−	−
*Erwinia persinia* 12	+	++	+	−	−	−	++	−
*Pantoeaaqqlomerans* 2	+++	++	++	+	−	−	−	−
*Pantoea aqqlomerans* 153	++	++	++	+	−	−	−	−
*Pantoea aqqlomerans* 43	+++	++	++	+	−	−	−	−
*Pantoea* sp. 287	++	++	++	+	−	−	−	−
*Pseudomonas orientalis* 24	−	+	−	−	+	+++	+++	−
*Pseudomonas synxantha* 283	−	−	−	−	−	+++	−	−

*^a^Lignin peroxidase activity in MEA-RBBR. LAC, laccase activity.*

### Bacterial Antifungal Compounds

To isolate antifungal metabolites from gut bacteria, dehydrated bacterial cultures were extracted using different solvents to determine the extraction efficiency. The results showed that methanolic extracts from *Ps. synxantha*, *Ps. orientalis*, *P. aqqlomerans* 2, *E. billingiae*, and *E. persicina* demonstrated the highest antifungal activities compared with other solvents (Table 4). The strongest antifungal activity was observed for *Ps. synxantha* and *P. aqqlomerans* 2 compared with other gut bacteria. Since *Ps. synxantha* was able to strongly inhibit almost all selected fungal species and could be a potential bacterium preventing fungal growth in the gut, further work along these lines will be continued.

**TABLE 4:** Effect of different solvents on the extraction efficiency of active compounds.

Bacteria	Solvent		
	Methanol	Ethyl acetate	Petroleum ether
*Pseudomonas synxantha* 283	+++++	++	−
*Psedomonas orientalis* 24	+	−	−
*Pantoea* sp. 287	−	−	−
*Pantoea aqqlomerans* 43	−	−	−
*Pantoea aqqlomerans* 153	−	−	−
*Pantoea aqqlomerans* 2	+++	−	−
*Erwinia billingiae* 32	+	−	−
*Erwinia persicina* 12	+	−	−

An estimated 280-mg crude methanolic extract from *Ps. synxantha* was re-extracted with silica and Sephadex gel column chromatographies using different solvent systems. Each fraction was examined with an agar diffusion to determine its antifungal activity and monitored with TLC. For the final purification of the active fraction, Sephadex column chromatography was used with a dichloromethane mobile phase. The purity of the active fraction was analyzed with preparative HPLC showing a single symmetrical peak at 250 nm with a retention time of 9.1 min. The dried pure compound was greenish-yellow, needle-crystalline, and soluble in chloroform, DMSO, and methanol but insoluble in water.

**FIGURE 5:**
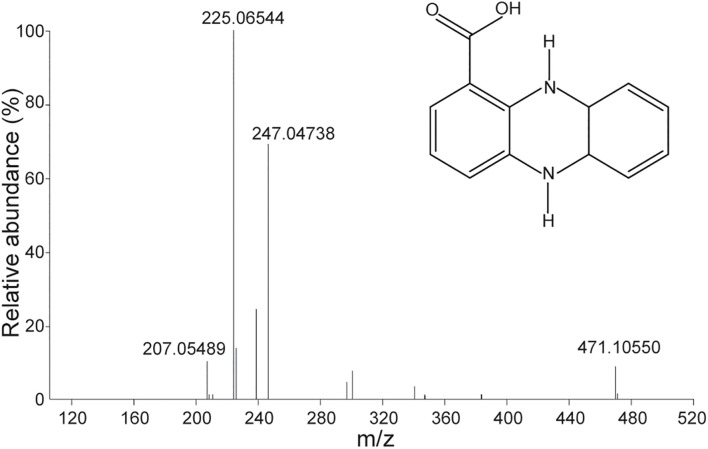
Mass spectra of antifungal compounds and the structure of phenazine-1-carboxylic acid (PCA).

Pure antifungal compounds were characterized by ESI-MS, ^1^H-NMR, and ^13^C-NMR. The molecular mass spectra demonstrated ion peaks at *m/z* 225.06544 [M + H]^+^ (base peak) and *m/z* 247.04738 [M + NA]^+^ (Figure 5). The ^1^H and ^13^C NMR spectral data of the compounds and their assignments are shown in Table 5. After NMR and mass spectrometry analyses, the compound of interest was determined to be phenazine-1-carboxylic acid.

**TABLE 5:** ^1^H and ^13^C NMR chemical shifts of phenazine-1-carboxylic acid and HMBC data in CDCL_3_, *δ*, ppm at 400 MHz.

Atom position	*δ*_H_ (*J* in Hz)	*δ*_C_	HMBC (H→C)
1		125.06	
2	8.53, dd (8.7, 1.1)	135.25	4, 1a
3	8.05, m	130.42	1, 4a
4	8.98, dd (7.1, 1.1)	137.57	2, 1a, COOH
1a	−	140.19	
4a	−	143.51	
5a	−	144.22	
8a	−	139.97	
5	8.35, dd (8.1, 1.8)	130.21	7, 8a
6	7.97, dd (6.6, 1.3)	131.88	5a, 8
7	8.01, m	133.36	5, 8a
8	8.29, dd (8.0, 1.8)	128.11	5a, 6
COOH		166.07	

### Minimum Inhibitory Concentration and Scanning Electron Microscopy

**FIGURE 6:**
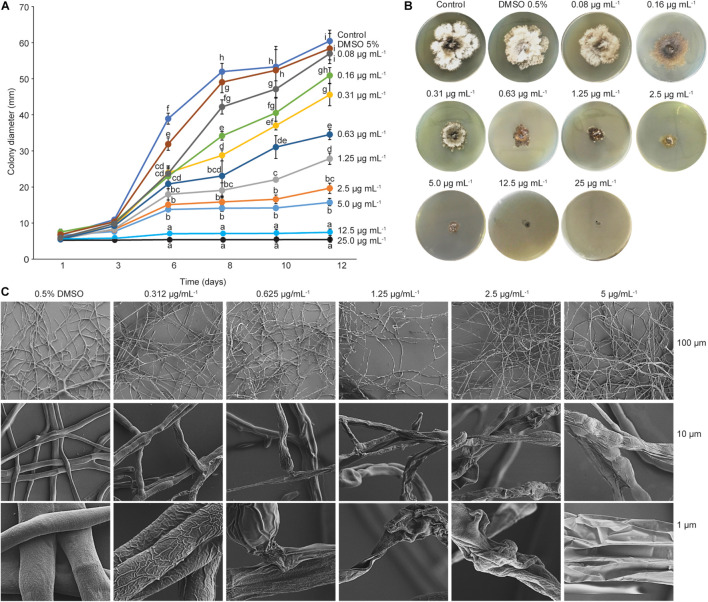
Minimum inhibitory concentration and scanning electron microscopy. **(A)** Minimum inhibitory concentration (MIC) of PCA. Graphic represents the effects of different PCA concentrations on fungal growth at different incubation time. Fungal growth decreased at a concentration of 0.08 mg ml^–1^ PCA, and complete inhibition of mycelial growth was observed at 25 and 12.5 mg ml^–1^. Different letters indicate significant differences determined by one-way ANOVA, followed by a Fisher PLSD *post-hoc* test (*p* < 0.05). **(B)** MIC assay after 12 days of incubation at 28°C. **(C)** Scanning electron microscopy (SEM) images of the effect of PCA against *D. sarmentorum*.

To determine the minimum inhibitory concentration, a filamentous fungus, *Dothiorella sarmentorum* #18, was chosen because of its faster growth on PDA. This species was also used during all purification steps to determine the antifungal activity of the fractions. Purified phenazine-1-carboxylic acid at an MIC of 12.5 mg ml^–1^ was able to clearly inhibit mycelial growth of *D. sarmentorum*. Serial dilutions of the compound showed that the effective doses at 50% and 80% were 1.25 and 5 mg ml^–1^, respectively (Figures 6A,B).

To further understand the effect of phenazine-carboxylic acid on fungal growth, mycelial growth was observed with SEM. Mycelia obtained from the edge of the *D. sarmentorum* colony growing in antifungal compound-free medium (control) produced hyphae with smooth surfaces (Figure 6C). With the addition of several concentrations of PCA ranging from 0.312 to 5 μg ml^–1^ into the medium, fungal hyphae lost smoothness depending on the PCA concentration. A high amount of PCA caused the failure to form a hyphal network thus inhibiting fungal growth.

### Origin of the Gut Bacteria

**FIGURE 7:**
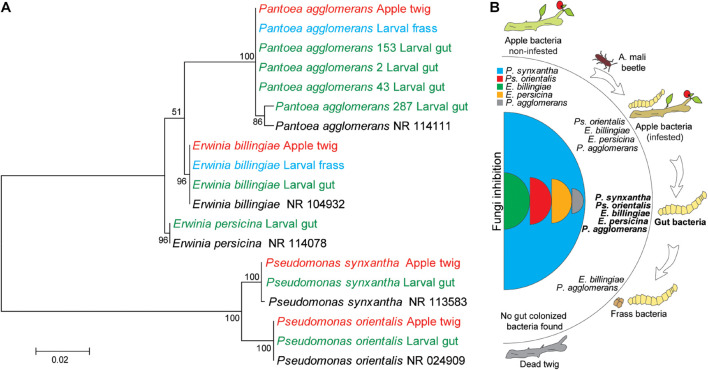
Identity analysis of the gut bacteria and possible route of bacterial transmission to larvae. **(A)** Clustering of bacterial species from the larval gut, apple twig-associated bacteria, and frass. The evolutionary history was inferred by using the maximum likelihood method based on the Tamura–Nei model. The percentage of replicate trees in which the associated taxa clustered together in the bootstrap test (500 replicates) is shown next to the branches. The evolutionary distances were computed using the Tajima–Nei method and are in units of the number of base substitutions per site. Distance scale represents the number of differences between the sequences. Evolutionary analyses were conducted in MEGA 7. Bacterial species in black, red, and green words relate to GenBank, apple twigs, and the gut bacteria, respectively. **(B)** Possible mechanism of transmission of tree bacteria to the larval gut.

After hatching, insect larvae can adopt bacteria either directly parentally or from the host during feeding. To understand the origin of gut bacteria, we compared 16S RNA sequences of the same species found in apple twigs and gut bacteria (Figure 7A). For this purpose, we analyzed bacteria from noninfested and infested twigs and larval frass. Since gut bacteria could be excreted, they could be found in larval frass. Sequence analysis and the neighbor-joining tree revealed that the gut bacterial gene sequences were identical to those of related apple bacteria (Figure 7A). *Erwinia billingiae*, *E. perscina*, *Ps. orientalis*, *Ps. synxantha*, and *P. agglomerans* specifically colonized non-infested twigs were still found in infested twigs but not in dead twigs. *Erwinia billingiae* and *P. agglomerans* were detected in the larval frass since these bacteria were highly abundant in the gut and thus could be excreted (Figure 7B).

## Discussion

In nature, many microorganisms interact with each other to coexist, and their ecological relationships range from parasitism to mutualism (Prosser et al., 2007). Depending on the species, insects have symbiotic associations with diverse and complex resident and transient microorganisms (Hooper and Gordon, 2001; Egert et al., 2003; Dillon and Dillon, 2004; Douglas, 2015). In previous work, the larval gut of the invasive wood-borer *A. mali* demonstrated a diverse bacterial community, but among them, the most abundant *Panthoea* spp. (99%), some *Erwinia* species, and *Pseudomonas* species, were able to degrade plant cell wall compounds (Bozorov et al., 2019a). The larval gut did not show any fungal species using either high-throughput sequencing or culture-dependent methods (Bozorov et al., 2019b). In this study, we explored the interaction between the gut bacteria of invasive wood-borer insect larvae and wild apple-associated fungi to elucidate the absence of fungi in the larval gut.

Fungi and bacteria can be transiently or permanently associated with the host and transmitted to the gut from their parents or the host (Feldhaar, 2011; Hammer et al., 2017). In contrast to the results of Zhang et al. (2018), who showed that *A. mali* larvae can be colonized by fungi and bacteria, our earlier report demonstrated the absence of fungi in the larval gut of *A. mali* (Bozorov et al., 2019b). However, both fungi and bacteria can colonize the congeneric *A. planipennis* (Mogouong et al., 2020). Hypothetically, the initial *A. mali* invasion of the Tianshan forests could have involved both bacterial and fungal species but eventually, competition by local microorganisms within the gut could have orchestrated the gut community, resulting in the elimination of fungal species from the gut. This could result in recompositing the larval gut microbiota to adapt to new environments for better insect survival. The diversity of the insect gut microbiota can be determined by the environmental habitat, diet, developmental stage, and phylogeny of the host (Yun et al., 2014). The gut microbiota could play an important role in the larval overwintering process at low temperature (Wang et al., 2017; Ferguson et al., 2018), and the climate of the natural habitat of *A. mali*, the Far East, Eastern China, and the Korean Peninsula, is not colder than that in Tianshan (Yang et al., 2017; Yao et al., 2020). The most abundant *Panthoea* bacteria of the larval gut with strong lygnocellulosic ability might provide sugars as energy resources and as cryoprotectants. During overwintering, insects accumulate sugars and polyols (Khani et al., 2007; Kostal et al., 2007) that can act as cryoprotectants and enhance cold hardiness for winter survival (Kost’al et al., 2001). However, to deeper understand the invasive insect larvae adaptation, it requires conducting additional *in vitro* experiments.

Both fungal and bacterial compositions varied in different twigs. Bacterial abundance was high in noninfested twigs, but fungal abundance was higher in infested twigs. This indicates that plant immunity reduced because larvae feed on the phloem of trees by creating serpentine galleries, thus preventing nutrient movement. The transition from twig infestation to twig death, thus, creates the growth condition for saprophytic wood decaying fungi (WDF) (Lundell et al., 2014; Park et al., 2020). Lignin is known to be extensively degraded by WDF and other fungi (Lundell et al., 2014; Park et al., 2020). Eversince, *A. mali* larvae specialized in less lignified phloem tissue compared with lignified xylem and that the gut bacteria of larvae degrade cellulose but not lignin (Bozorov et al., 2019b). Moreover, the lignin composition and content vary between xylem and phloem (Lourenço et al., 2016). Wood-decaying saprophytic fungi are the majority of species belonging to Basidiomycota and Ascomycota (Lundell et al., 2014). Bacteria and WDF must interact with each other to coexist, although WDF are well known for being highly competitive (Boddy, 2000). This is consistent with our result that bacterial composition was highly reduced in dead twigs. Saprophytic fungi can be inhibited by symbiotic bacteria when co-cultured together on artificial medium (Figure 4B) but in nature, dead twigs cannot be a food source for bacteria. This indicates that tree-associated bacteria found in this study could be symbiotic bacteria growth of which rely on relationship with the living plant, but in dead twigs bacteria might not grow because of the interrupted symbiotic link. Likely, bacterial species from non-infested trees must be transmitted into the larval gut because the originated gut bacteria were reduced or not found in the infested twigs (Figure 7B). Additionally, fungal isolates could also be transmitted into the *A. mali* gut, as reported by Zhang et al. (2018), but our study showed that this might not have occurred in the Tianshan forests (Bozorov et al., 2019b). Probably, presence of gut antagonistic bacteria might prevent fungi colonization of the gut. However, additional *in vitro* experiments are required to clarify bacterial communities of insect host trees of natural habitat and invaded area.

The current study demonstrated that all gut bacteria were able to antagonize tree-associated fungi with different inhibition levels. However, *Ps. synxantha* inhibited almost all fungal species with the highest inhibition rate. Possibly, this bacterium might play as key competitor against fungi in the gut. Apparently, fungi also had a low composition and abundance in larval frass that might be caused by the presence of antifungal compounds produced by the gut bacteria. However, no *Pseudomonas* species were detected in the larval frass. In contrast to *Pantoea aqqlomerans* and *E*. *billingiae* that were detected in the larval frass, it could be that *Pseudomonas* species might be localized in one of the upper compartments of the gut, such as the foregut or midgut. Chen et al. (2018) demonstrated that a more diverse microbial community was found in the foregut than in the midgut and hindgut in silkworms (*Bombyx mori*). However, experimental validation of bacterial species localization by gut compartments is required.

Several studies have reported the antagonistic ability of *Pseudomonas* species against fungi by the production of various types of antifungal metabolites (Jayaswal et al., 1993). Phenazine carboxylic acid (PCA) is one of the antifungal compounds produced by *Pseudomonas chlororaphis, Ps. Fluorescens*, and *Ps. protegens* that is able to kill some lepidopteran larvae (Flury et al., 2017) but the current study showed that PCA produced by *Ps. synxantha* was not lethal to *A. mali* larvae. Likely, PCA might prevent fungal colonization in the gut, which likely might explain the absence of fungi in the larval gut. This contrasts with the results of Zhang et al. (2018), who showed that both bacteria and fungi were found in *A. mali* larvae, including one *Pseudomonas* sp., but identification in species level was not clear. However, this species could be *Ps. orientalis* identified in our study as possessing weak inhibition of some apple-associated fungi. It was reported that these bacteria produce various types of antifungal compounds, such as pyoverdine, safracin, and phenazine, which are effective against bacteria and fungi (Kron et al., 2020). Moreover, *Ps. orientalis* has been isolated from most coleopteran insects due to its encoded cellulolytic enzyme involved in terpene transformation of plant resin compounds (Rojas-Jiménez and Hernández, 2015); this is consistent with our result that *Ps. orientalis* plays a role in cell-wall degradation (Bozorov et al., 2019b).

Other gut bacteria, such as *Panthoea* and *Erwinia*, also showed average antagonistic abilities against some apple-associated fungi despite their strong lignocellulolytic activities (Bozorov et al., 2019b). The most abundant gut bacteria, *P. aqqlomerans*, also had antifungal activity but was not able to inhibit all endophytic and saprophytic fungi. However, its antagonistic ability is reported in the literature (Iturritxa et al., 2017; Thissera et al., 2020). It was reported that *E. billingiae* also has antifungal ability against pathogenic *Heterobasidion annosum*, *Armillaria mellea*, and *Fusarium circinatum* fungi infecting *Pinus radiata* trees (Mesanza et al., 2016; Iturritxa et al., 2017), and *E. persicina* has activity against *A. alternata* (Goryluk-Salmonowicz et al., 2016). It is likely that bacterial species within the genus could produce similar antifungal compounds, such as herbicolin, pulicatin, and pyrrolnitrin (Greiner and Winkelmann, 1991; Chernin et al., 1996; Thissera et al., 2020).

We analyzed wild apple-associated bacteria, and some of them were transmitted to the gut of *A. mali* larvae. Among them, *Ps. synxantha* produced the antifungal phenasine carboxylic acid compound that strongly inhibited apple-associated fungal species; thus, it might inhibit fungal colonization in the larval gut. Other gut bacterial species, *Panthoea* and some *Erwinia* species, participated in plant cell wall cellulose degradation. Taken together, in the invaded area, insect larvae adopt selectively the apple-associated fungi and bacteria to build its own gut microflora. The gut bacterial community might participate in intensive plant cellulosic degradation to provide an energy source for the larvae to better adapt and survive in new regions.

## Data Availability Statement

The original contributions presented in the study are included in the article, further inquiries can be directed to the corresponding author/s.

## Author Contributions

TB wrote the manuscript and prepared all the tables and figures. TB, ZT, and GK contributed to the compound extraction and purification. ZT contributed to the compound analysis and structural determination. TB and YG contributed to fungi analysis. TB, DZ, and HS contributed to the manuscript writing and corrections. TB and DZ supervised the experiment and manuscript writing. All authors reviewed the manuscript.

## Conflict of Interest

The authors declare that the research was conducted in the absence of any commercial or financial relationships that could be construed as a potential conflict of interest.

## Publisher’s Note

All claims expressed in this article are solely those of the authors and do not necessarily represent those of their affiliated organizations, or those of the publisher, the editors and the reviewers. Any product that may be evaluated in this article, or claim that may be made by its manufacturer, is not guaranteed or endorsed by the publisher.
